# Mucocutaneous Manifestations of Behçet's Disease

**DOI:** 10.3389/fmed.2020.613432

**Published:** 2021-02-01

**Authors:** Koichiro Nakamura, Yuichiro Tsunemi, Fumio Kaneko, Erkan Alpsoy

**Affiliations:** ^1^Department of Dermatology, Saitama Medical University, Saitama, Japan; ^2^Institute of Dermato-Immunology and Allergy, Southern Tohoku General Hospital, Fukushima, Japan; ^3^Department of Dermatology and Venereology, School of Medicine, Akdeniz University, Antalya, Turkey

**Keywords:** Behçet's disease, erythema nodosum-like lesion, papulopustular lesion, oral ulcer, genital ulcer

## Abstract

Behçet's disease (BD) is a chronic, relapsing, systemic inflammatory disease with clinical features showing mucocutaneous lesions involving the ocular, articular, and further miscellaneous organs. Mucocutaneous manifestations, one of the most characteristic signs of BD, have been most commonly observed upon onset or at any disease stage and are exceptionally important in its diagnosis. Given the lack of specific diagnostic laboratory tests for BD, diagnosis has been based on clinical findings. All diagnostic criteria published have thus far relied heavily on mucocutaneous manifestations, particularly oral ulcers (OU), genital ulcers (GU), cutaneous lesions, and pathergy test positivity. Worldwide, OU, GU, cutaneous lesions, and ocular and articular manifestations have been the most common symptoms, with erythema nodosum (EN)-like lesions and papulopustular lesions being the most prevalent cutaneous manifestations. While majority of the patients worldwide have reported OU as the most frequent symptom upon disease onset, GU, and EN-like lesions have also been identified upon onset. Considering that mucocutaneous symptoms precede severe organ involvement in most patients, familiarity with such symptoms is imperative for early diagnosis and prevention of potentially serious organ involvement through appropriate management.

## Introduction

Behçet's disease (BD) is a chronic, relapsing, systemic inflammatory disease of unknown etiology that is clinically characterized by oral ulcers (OU), genital ulcers (GU), cutaneous lesions, and ocular symptoms.

However, specific clinical types with vascular, gastrointestinal, and central nervous system involvement have also been reported (1–4). Given the lack of definitive diagnostic laboratory and histopathological tests for the disease, diagnosis has solely been based on clinical findings. The various diagnostic criteria used in diagnosing this disease have commonly included mucocutaneous lesions, particularly OU, GU, cutaneous lesions, and pathergy test positivity (5). The most extensively utilized set of diagnostic criteria has been established by the International Study Group for Behçet's Disease criteria, among three of the five findings (OU, GU, cutaneous lesions, ocular involvement, and pathergy test positivity) comprise mucocutaneous lesions including OU, GU, and cutaneous lesions (6). The Japanese Diagnostic Criteria, one of the most sensitive and specific criteria, have been used particularly in Far Eastern countries (2), among which three of the four major criteria (OU, GU, cutaneous lesions, and ocular involvement) include mucocutaneous symptoms.

Mucocutaneous lesions have been the most common symptoms upon onset or at any stage of BD. Accordingly, OU (92–100%) and GU (57–93%), cutaneous lesions (38–99%), and ocular (29–100%) and articular manifestations (16–84%) have been the most common symptoms observed globally, with erythema nodosum (EN)-like lesions (15–78%) and papulopustular lesions (PPL) (28–96%) being the most frequently documented cutaneous manifestations. While OU have been the most frequently observed symptoms upon disease onset in majority of the patients worldwide, GU and EN-like lesions have also been reported at onset (7). Previous studies have demonstrated that a significant proportion of patients, only present with mucocutaneous symptoms (8, 9). However, the absence of major organ involvement in the early years of BD does not suggest a mild prognosis, particularly for young male patients. Considering that in most instances, mucocutaneous lesions appear before serious organ involvement develops. Therefore, familiarity with the mucocutaneous spectrum is imperative for early diagnosis and treatment, which would prevent most of the hazardous consequences of BD.

The current review will discuss the latest evidence regarding mucocutaneous manifestations of BD.

## Oral Ulcers

OU, one of the major initial signs of the disease, have been found in 97–99% of patients according to data obtained by the International Study Group for Behçet's Disease from several countries (6). OU typically occurs at an average of 7 years before the diagnosis. Bang et al. prospectively followed 67 cases with recurrent aphthous stomatitis (RAS) and observed that 35 (52.2%) patients developed BD symptoms after an average of 7.7 years. Similarly, they found the annual frequency of the lesions to be 9.8 in progressive patients (10).

Patients with OU initially present with a pseudo-membrane in the center surrounded by a reddish halo, shortly reaching the central ulcer. OU generally appear on the lips, buccal mucosa, gingiva, and tongue, with lesions occasionally developing on the palate. Albeit rarely, OU may occur on the tonsil and pharynx and cause considerable discomfort and resistance to conventional treatment modalities.

**Figure 1:**
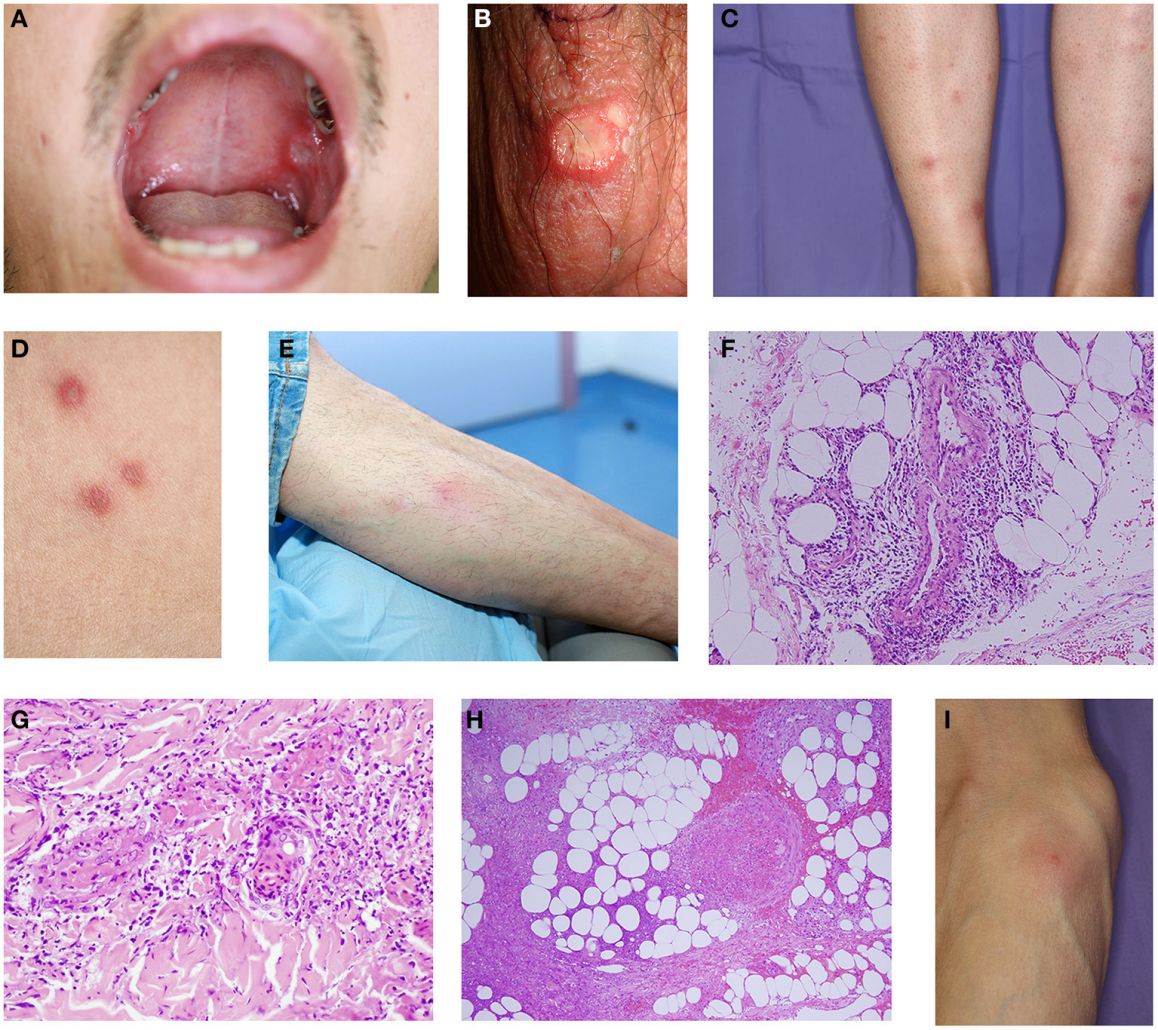
**(A)** Oral ulcer on the buccal mucosa with rolled borders and grayish necrotic base. **(B)** Genital ulcer with yellowish necrotic tissue on the scrotum. The genital ulcer is deep and surrounded by erythema. The scrotum is the site most commonly involved in the male. **(C)** Erythema nodosum-like lesions on the lower leg. A painful, reddish erytemanous nodule is detected in the pretibial lesion. **(D)** Papulopustular folliculitis are sterile pustules on the erythematous base of the trunk. **(E)** Superficial thrombophlebitis is erythematous tender subcutaneous nodules arranged in a linear fashion on the leg. **(F)** Histological feature of erythema nodosum-like lesion includes a dense neutrophil infiltrate around the vessels in the subcutaneous tissue. **(G)** Histological feature of papulopustular lesion includes a leukocytoclastic vasculitis with neutrophil infiltrate in the dermis. **(H)** Histology of thrombophlebitis shows vascular thrombus and lymphocytic infiltrate in a subcutaneous vein. **(I)** Skin pathergy reaction is a pustule formation at the needle site after 24 h on the forearm. **(A,F,H,I)** are derived from Nakamura et al. (3, 4).

Minor OU are small and shallow ulcers, usually 3–6 mm (<10 mm) in diameter. Although minor OU, seen in about 80% of patients, heal rapidly with no scarring, OU regress and recur repeatedly over a prolonged period. Major OU are large and deep ulcers, having diameter more than 10–30 mm and have severe pain (Figure 1A). While major OU are morphologically similar to minor OU, they are larger (>1 cm), deeper, and more painful. Moreover, such ulcers last longer and often heal with scarring and tissue loss. Notably, major OU, which occur frequently in patients with BD, have been one of the important parameters in distinguishing BD from RAS. In a comparative study of 1,643 and 3,527 patients with RAS and BD, respectively. Oh et al. reported that major OU were significantly more common and affected more oral mucosa regions in patients with BD (11).

Although OU alone have limited diagnostic value, such value increases in the presence of GU, uveitis, etc. However, OU have been the most common reasons for referral to the outpatient clinic and are one of the most active symptoms of BD, with recurrent periods of exacerbation and remission. Accordingly, a cross-sectional multicenter study, observed OU activity in 65.1% of the study group, and found that OU activity was associated with non-immunosuppressive use in both genders, as well as early disease in males and resistant cases in females (12). As such, suppression of OU activity is a crucial and common practice in the management of BD.

The appearance of OU often causes quality of life (QOL) disturbances. Accordingly, a study examining the 1-year prevalence of OU and its association with oral health-related QOL in patients with BD and general population found that those with BD who had OU (odds ratio, 6.2; *n* = 529) had more disturbed QOL compared to general population without OU (odds ratio, 1.0; *n* = 1,097) (13).

## Genital Ulcers

GU, another characteristic feature of BD, may serve as the initial sign of the disease. Although the appearance and course of GU are similar to those of OU, they are generally deeper and less often recurring (Figure 1B). Given that deep-seated ulcers may heal with scarring, scars of previous lesions should be investigated in patients suspected to have BD, despite the absence of GU at that time. Mat et al. who investigated the frequency of scarring in GU (14), found that 89% of large ulcers (≥1 cm) and 49% of small ulcers (<1 cm) healed with scarring in male patients. Meanwhile, the rate of scar formation in female patients with large and small GU was 100 and 40.5%, respectively. The aforementioned study observed that ulcers located in the labium minus and vestibule can heal without scarring. Therefore, scars from previous lesions should be investigated apart from active lesions in the diagnosis of BD.

Approximately 90% of GU in men develop on the scrotum, with lesions rarely locating on the glans and corpus penis. In women, the most common localization is the labium, although they develop in the vulva, vagina, and even cervix. Bloody-purulent discharge, pain, and dyspareunia may be observed due to its location in the vagina. Moreover, vulval ulcers can occasionally lead to tissue loss in the labia. Although rare, deeply located ulcers may fistulize into the bladder, urethra, and rectum. Furthermore, reports have been shown perianal region and inguinal fold localization in both sexes (15).

In summary, GU are one of the three symptom complexes originally characterizing the disease. Compared to OU, GU are more specific for the diagnosis of BD. The International Study Group for Behçet's Disease compared the data of 914 patients with Behçet's disease from 12 centers in 7 countries with those of controls in the same centers. In a new diagnostic criterion, the sensitivity and specificity of each BD symptom were determined and relative value and expected value were calculated. Of all BD symptoms, GU had the highest relative value and expected value (6). The presence of recurrent OU and GU are strongly evocative of BD, especially in endemic regions.

## Cutaneous Lesions

EN-like lesions, PPL and superficial thrombophlebitis represent the most characteristic cutaneous lesions of BD. Moreover, cutaneous lesions are among the first symptoms of BD and often show a course with recurrent attacks (7). Therefore, cutaneous lesions need to be diagnosed in the early stages of BD. One study showed that among patients with BD in Japan, 88.8% (513 of 578 patients) exhibited cutaneous lesions (16).

### Erythema Nodosum (EN)-Like Lesions

EN-like lesions are painful, tender, oval, erythematous nodules that often develop on the legs (Figure 1C) and are more common in females. Although most frequently observed in the lower extremities, they can also be located in other body parts, such as the gluteal region, upper extremities, face, and neck. The lesions regress within 2–3 weeks with local pigmentation and sometimes scaling, especially in dark-skinned individuals. Several patients with EN-like lesions have accompanying systemic symptoms, such as joint symptoms, malaise, and fever.

Although EN-like lesions are characteristic of BD, these lesions are not specific to the disease. Thus, differentiating between EN-like lesions of BD and classic EN, which is caused by bacterial or viral infections, such as streptococcal pharyngitis, is necessary. Other differentiate conditions include EN accompanied by ulcerative colitis, Sweet's syndrome, and Crohn's disease. A skin biopsy is useful to confirm the inflammatory and vascular changes seen in EN-like lesions of BD.

### Papulopustular Lesions

PPL, observed in 65–96% of the cases, have been the most common cutaneous manifestation of the disease. They are characterized by folliculitis or acne-like sterile papulopustules on an erythematous base (Figure 1D). Typical lesions appear as papules that develop pustules within 24–48 h and are often localized in the trunk, lower extremities, and face (17). Moreover, PPL have been frequently reported in patients with arthritis (18).

BD is usually diagnosed between the ages of 20–30 during which acne vulgaris is also relatively common. Distinguishing between PPL in BD from acne vulgaris is extremely important in patients diagnosed with BD in the presence of PPL. We believe that follicular PPL is not specific to the disease. PPL in BD include follicular and non-follicular lesions. Follicular lesion of BD sometimes includes a hair follicle, namely a pustule containing hair designed as a folliculitis. However, Boyvat et al. have observed neutrophilic vasculitis in cases with non-follicular PPL (19) and this observation supports the notion that non-follicular PPL is more specific to the clinical characteristic feature of BD (Figures 1D,G). Therefore, considering only non-follicular PPL would be more appropriate. Again, localization in non-seborrheic areas, such as the lower extremities, can be more specific for BD and should be preferred as a biopsy site.

### Superficial Thrombophlebitis

Superficial thrombophlebitis is a palpable induration along the course of a vein, mainly in the legs (Figure 1E) that occurs more frequently in male cases and most frequently affects the vena saphena magna. This condition may present clinically as superficial migratory thrombophlebitis. Clinically distinguishing between superficial thrombophlebitis and EN-like lesions may be difficult when a short vein segment is involved. Nonetheless, ultrasonography can help differentiate both conditions in addition to clinical examination, with dermal ultrasonography, revealing a hyperechoic pattern in EN-like lesions, but a hypoechoic pattern in superficial thrombophlebitis.

Although superficial thrombophlebitis can be detected during dermatological examination, it indicates vascular involvement and closely associated with deep vein thrombosis and dural sinus thrombosis (20). Close follow-up of cases with superficial thrombophlebitis should therefore be appropriate in terms of vascular involvement.

### Histopathology of Cutaneous Lesions

Histologically, early-stage EN-like lesions are characterized by septal panniculitis accompanied by a marked neutrophilic infiltrate, particularly around blood vessels in the upper and lower dermis, and extravascular leakage of red blood cells (Figure 1F). Lymphocytic infiltrates predominate in addition to neutrophils in the late stage. Occasionally, a vascular thrombus can occur as inflammatory changes. The existence of neutrophilic perivascular infiltration, sometimes vasculitis with neutrophil infiltration, is a pathological finding of EN of BD, which is an important feature differentiating from classical EN.

In histopathological examination of papulopustular lesion, pustules exhibit neutrophilic infiltrates around the hair follicles and just below the epidermis. Neutrophils also infiltrate around the blood vessels in the dermis. One study showed that leukocytoclastic vasculitis in 10 (43.5%) of 23 patients with pustular lesions (19). Meanwhile, Alpsoy et al. reported that 12 (70.5%) of 17 patients with BD exhibited 11 leukocytoclastic vasculitis and 1 lymphocytic vasculitis (21). Moreover, Ilknur et al. reported that 27.5% of patients with BD experienced vasculitis, whereas none of the controls showed the same (22). However, Ergun et al. reported that PPL do not affect the development of vasculitis (23). Finally, Jorizzo et al. reported the importance of histologic changes in non-follicular PPL for the diagnosis of BD (24). Given that vascular histopathological changes may be specific for PPL, we strongly suggest considering the presence of leukocytoclastic vasculitis or neutrophilic vascular reaction in the histology of non-follicular PPL as a diagnostic criterion (21) (Figure 1G).

Histologically, stenosis and thrombus formation have been observed in the lumen of a superficial subcutaneous vein along with neutrophil and lymphocytic infiltrates of superficial thrombophlebitis (Figure 1H). The Chapel Hill Consensus Conference indicated that BD is involved in variable-sized vessel vasculitis (25). Another study showed that among 2,319 patients, 14.3% had vascular involvement, while 53.3% had superficial thrombophlebitis, indicating that superficial thrombophlebitis was the most common feature of vascular change (26).

### Other Cutaneous Lesions

Other common and well-known skin manifestations of BD include extragenital ulcers and Sweet's syndrome-like lesions.

Extragenital ulcers, which are clinically similar to aphthous lesions and are one of the most specific symptoms of BD, are deep-seated, punched-out ulcers with erythematous and edematous edges and a yellow necrotic base. Extragenital ulcers follow a recurrent course, can usually result in scarring, and can be localized to the legs, armpits, breast, neck, toes, inguinal region, and neck. Meanwhile, Sweet's syndrome-like lesion is a painful reddish plaque usually observed in the face, neck, and extremities and rarely associated with BD. Other lesions (e.g., pyoderma gangrenosum-like lesions, erythema multiforme-like lesions, pernio-like cutaneous lesions, palpable purpura, Henoch-Schönlein purpura, bullous necrotizing vasculitis, subungual infarctions, hemorrhagic bullae, furuncles, abscesses, and acral purpuric papulonodular lesions) have been limited to case reports. Histologically, neutrophilic infiltrates have been observed in the skin of all such lesions and are therefore referred to as neutrophilic dermatoses.

## Skin Pathergy Test

A pathergy test determined the hypersensitivity reaction after a needle puncture (Figure 1I), with positivity being defined as the development of an erythematous papule or pustule around the injection site 24–48 h after intradermal puncture of the skin. While ~50% of the patients in the Middle and Near East have pathergy test positivity, ~ <30% in Japan have the same. Numerous factors affect the positivity rate of skin pathergy tests, including the thickness of the needle used, sharpness or blunt tip of the needle, number of punctures, method of application, disinfection of the test site with an antiseptic, etc, which might explain the differences in pathergy test positivity among these countries. A positive result to a pathergy test is a major criterion in the International Criteria for Behçet's Disease and International Study Group for Behçet's Disease and is an important manifestation for early diagnosis.

## Conclusion

We hereline discussed clinical features of mucocutaneous lesions observed in BD. Accordingly, we highlight that mucocutaneous lesions precede the diagnosis of BD. Moreover, given that patients can show variable symptoms during their course, excluding other disease is always necessary to ensure correct diagnosis. Characteristic feature of BD, such as OU, GU, EN-like lesions, PPL, superficial thrombophlebitis, and skin pathergy test are manifestations that can be used for early diagnosis. Considering that patients often show poor QOL, early diagnosis and treatment initiation with careful observation of the course is favorable.

## Author Contributions

KN, YT, FK, and EA: writing and revision of the manuscript. KN, YT, and EA: acquisition of clinical data. EA: conception and design and study supervision. All authors contributed to the article and approved the submitted version.

## Conflict of Interest

The authors declare that the research was conducted in the absence of any commercial or financial relationships that could be construed as a potential conflict of interest.
